# Development and validation of an online tool to assess perceived portion size norms of discretionary foods

**DOI:** 10.1038/s41430-023-01290-y

**Published:** 2023-05-22

**Authors:** Qingzhou Liu, Leanne Wang, Siyi Guo, Margaret Allman-Farinelli, Anna Rangan

**Affiliations:** 1 School of Life and Environmental Sciences, Faculty of Science, The University of Sydney, Sydney, NSW 2006 Australia; 2 Charles Perkins Centre, The University of Sydney, Sydney, NSW 2006 Australia; 3 Discipline of Nutrition and Dietetics, Susan Wakil School of Nursing and Midwifery, Faculty of Medicine and Health, The University of Sydney, Sydney, NSW 2006 Australia

**Keywords:** Nutrition, Public health

## Abstract

**Background:**

Perceived portion size norms (typical perception of how much of a given food individuals choose to eat at a single occasion) may have shifted towards larger sizes due to the ubiquity of large serving sizes. However, there is a lack of validated tools to assess such norms for energy-dense and nutrient-poor discretionary foods. This study aimed to develop and validate an online tool to examine the perceived portion size norms of discretionary foods.

**Methods:**

An online image-series tool of 15 commonly consumed discretionary foods was developed, with eight successive portion size options included for each food. Using a randomised-crossover design, adult consumers (18–65 years) completed the validation study in a laboratory session (April-May 2022) by reporting their perceived portion size norms for each food twice, once based on food images on a computer and another time based on equivalent real food portion size options at food stations in the laboratory. Agreement between methods for each test food was examined using cross-classification and intra-class correlation (ICC).

**Results:**

A sample of 114 subjects were recruited (mean age 24.8 years). Cross-classification indicated >90% of selections were matched in the same or adjacent portion size option. ICC was 0.85 across all foods, demonstrating a good level of agreement.

**Conclusion:**

This novel online image-series tool developed to examine perceived portion size norms of discretionary foods showed good agreement with equivalent real food portion size options and may be valuable to investigate perceived portion size norms of common discretionary foods in future studies.

## Introduction

Perceived portion size norms, described as a typical perception of how much of a given food individuals choose to eat at a single eating occasion [1, 2], have a key role in food consumption and portion control behaviours [3, 4]. This norm may have been distorted towards larger sizes due to the ubiquity of large serving and package sizes currently available in the food environment [5, 6]. Recommended intakes from dietary guidelines may be considered “too small” as large servings are now perceived as the new “normal” [7]. This is especially concerning for discretionary foods, described as foods and drinks that are high in saturated fats, added sugars, added salt and/or alcohol and should be consumed sometimes and in small amounts [8]. Unconscious overconsumption of discretionary food can result in excessive energy intake, lower diet quality, and the development of obesity and chronic diseases long term [9, 10]. Reducing the upshifted portion size norm has therefore been highlighted as one potential strategy to tackle this trend and empower consumers to select more appropriate portion sizes [5, 11].

Different types of perceived portion size norms have been identified in previous research [1, 2]. For example, individuals might be guided by social norms of portion sizes (that is, beliefs about how much others expect them to eat) when dining out with a group, while personal norms of portion sizes (that is, beliefs about how much to eat according to oneself) may be more salient when eating alone in a home setting [1, 12]. Haynes and colleagues have proposed the ‘norm range model’, suggesting portion sizes within but at the lower end of the perceived normal range may nudge lower intakes unconsciously, whereas portion sizes reduced beyond that range are likely to result in additional consumption [3]. Nevertheless, a clear understanding of the range of perceived portion size norms across commonly consumed discretionary foods has yet to be established [10].

Various assessment tasks have been used to investigate the perceived portion size norm, including self-selected portion size tasks based on provided portion size options [13–15], normality judgement tasks using computer-based images [3], and an estimation of the number of portions contained in a package or container [16]. However, there is a lack of consistency and validation in terms of food presentation and the number of portion size options provided. Many tools for assessing perceived portion size norms using real foods only provided one single size option. This may influence the accuracy of portion size norm measures, given that the unit bias and social desirability bias have been consistently demonstrated [12, 17]. For example, individuals tend to rely on the size of a single food unit when making portion size decisions and may consider the displayed amount of food to be the socially expected portion size [12, 17, 18]. Although online image-based tools tend to provide a broad range of portion size options, they are not commonly pre-piloted nor validated in the population of interest [2, 19]. To minimise the interaction between serving size exposure and potential bias when selecting perceived portion size norms, careful consideration of food selection, presentation, as well as the number and range of options is required [2, 12, 19, 20].

To ensure the accuracy of outcome measures, assessment tools specifically designed to examine the perceived portion size norm are needed. A series of computer-based food images appears to be a promising alternative to using real foods in the estimation of perceived portion size norms due to a lower respondent burden and higher accessibility to a larger population [19, 21]. Therefore, the aim of current study was to develop and validate an online image-based tool to examine the perceived portion size norms (that is, the amount that is perceived to be their normal portion size) of discretionary foods among Australian consumers.

## Methodology

### Study overview

An online tool was developed to investigate the perceived portion size norm of commonly consumed discretionary foods in Australia. Image-series of 15 discretionary foods showing different portion sizes was validated against identical real food portion size options. Using a within-person crossover design, participants attended an in-person laboratory session to complete the validation study. During the session, participants were requested to select what they perceive to be their portion size norm for each test food twice; once based on food images on the computer screen, and the other time based on equivalent real food options displayed at the food stations. The order of completion of each method and the presentation order of test foods were randomised.

### Tool development

#### Selection of test foods and portion sizes

Based on the latest national nutrition survey [22, 23], a variety of commonly consumed and readily available discretionary foods were selected for inclusion; sweet snacks (M&Ms, chocolate bars, chocolate blocks, and sweet biscuits), cakes (layered cake, caramel slices, muffins, and banana bread), savoury snacks (savoury biscuits and potato crisps), fast foods (pizza, nuggets, and hot chips), and sugary carbonated drinks (cola, in glasses or cups, and in bottles or cans) (Table 1) [24].

**Table 1:** Nutrition information^a^ (per 100 g) of discretionary foods and drinks included in this study (*n* = 15).

	Nutrition information per 100 g				Portion size options, range	
	Energy (kJ)	Saturated fat (g)	Sugar (g)	Sodium (mg)	Energy (kJ)	
					Min	Max
M&Ms	2020	12	67	61	141	3636
Chocolate block	2250	19	56	82	248	3060
Chocolate bar	1890	8	58	125	189	2646
Sweet biscuits	1940	12	30	260	155	1900
Caramel slice	1850	17	42	193	370	3885
Layered cake	1361	3	33	320	463	5090
Muffin	1552	4	30	344	186	4734
Banana bread	1212	2	25	360	376	3515
Savoury biscuits	2060	5	1	685	144	3584
Crisps	2290	2	1	556	229	4030
Pizza	1044	4	2	550	647	5250
Nuggets	1104	6	0	740	221	5300
Hot chips	944	4	0	228	236	2710
Cola cup/glass	180	0	11	10	135	1080
Cola bottle/can	180	0	11	10	225	1080

^a^Based on the nutrition information panel on food packages (if available) and the Australian Food Composition Database [24].

Eight portion sizes in increasing size were included for each food (except for drinks in bottles or cans where six options were included). Portion size literature suggests that presenting a range of serving size options may assist with portion size estimations [20] and an even number of options helps to avoid the temptation of choosing the centre image [20, 25]. The portion size range and options were carefully selected. For each food, the lower and upper limit of portion size options were guided by searches of available package sizes available in chain supermarkets and fast-food outlets, the median and percentiles of typical portion sizes from previous literature [22], and feedback from our pilot study (March 2022). One of the three middle portion sizes (images 3–5) is an estimate of the typical (median) portion sizes based on the latest national nutrition survey [22]. Commonly available packages and/or a credit-card-sized marker were included in portion size options to reflect popular size options. More detailed criteria used to develop the portion size options and the corresponding portion size weights and energy content are available in supplementary materials (Appendix 1).

#### Tool set-up in Qualtrics

**Fig. 1:**
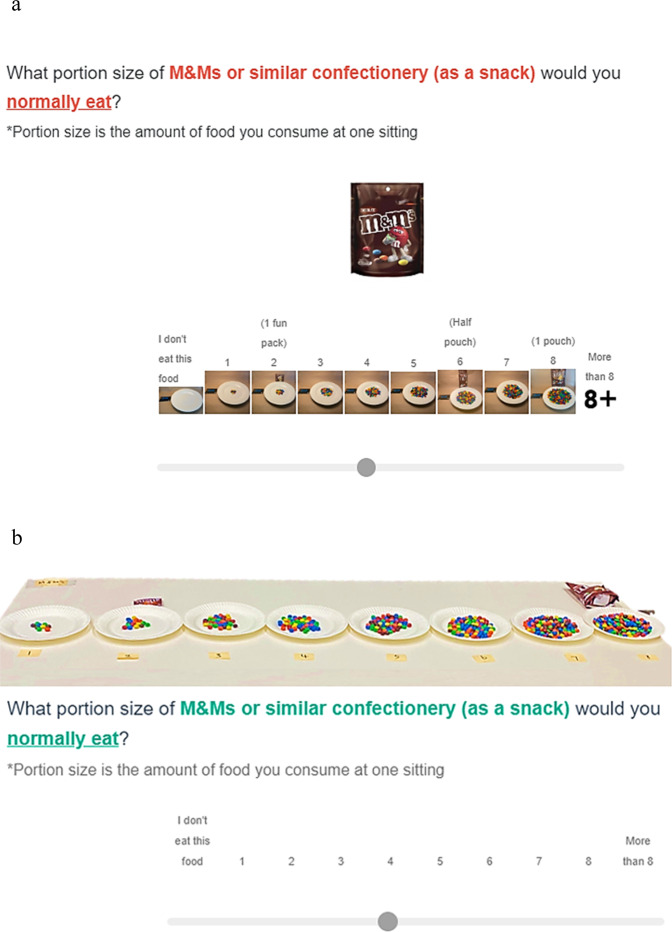
Survey question examples. **a** For food images section of the online tool. The eight successive images corresponding to the sliding scale question, labelled from smallest ‘1’ to largest ‘8’ and additional selections of ‘0 – I do not eat this food’ and 9 – greater than the largest option displayed’. Participants were instructed to select the closest match of their perceived portion size norms based on food images on the screen. **b** For equivalent real food portion size options of the online tool. The provided portion size options and questions were identical between the two methods. Participants were instructed to select the closest match of their perceived portion size norms by observing real food portion size options at the food stations.

The online tool was developed using Qualtrics (Qualtrics, Provo, UT, USA, 2022), an online survey development software. Image-series for all test foods were included in the food image section. In this section, the eight successive images were displayed to correspond with the sliding scale question, ‘What portion size of (the test food and setting; for example, sweet biscuits as a snack) would you normally eat?’. The sliding scale was labelled from smallest ‘1’ to largest ‘8’ plus the additional selections of ‘0 – I do not eat this food’ and ‘9 – greater than the largest option displayed’ (Fig. 1a). Participants were instructed to move the marker to their corresponding or nearest perceived portion size norm, which would become enlarged for easier viewing. The JavaScript code was based on Embling et al.’s image carousel [21]. An image of the original package (that is, how the food is typically sold) was used as the cover photo to orient the participants to each food.

The real food section consisted of the questions and sliding scale identical to the food image section as described above but without any food images (Fig. 1b). Participants answered each question in this section by observing the labelled food portion size options (weighed to the nearest gram by researchers) presented at each of the food stations in the laboratory room.

The presentation order of the food image and real food section, and the order of the test foods within these sections were randomised using a built-in randomiser in Qualtrics. The tool was pilot tested in the target population (March 2022) and minor modifications were made to improve usability. Further details of study and tool design, as well as the preparation process for food images and real food portion size options are attached as Supplementary material (Appendix 1).

#### Demographic section

The demographic section collected information on participants’ gender, age, self-reported height and body weight, postcode of home address, usual physical activity level (PAL), and confidence in their cooking skills. PAL was estimated using the physical activity factor and classified as sedentary, lightly to moderately active, very to extremely active [26, 27]. Confidence of cooking skills was assessed as a marker of food literacy [28] using a validated Likert scale [29].

### Participant recruitment and study procedure

A convenience sample of university staff and students was recruited through online advertisements and the distribution of physical flyers. An online screener questionnaire excluded participants who did not meet the following criteria: living in Australia, aged between 18–65 years, fluent in English, no current or previous diagnosis of an eating disorder, and who were able to attend an in-person laboratory session.

Participants attended the in-person session at a university campus in Sydney (April to May 2022). Instructions were provided upon arrival; participants were requested to complete the online tool individually using a laptop, and following the prompts at the start of each section (based on either food images on computer screen or real foods at food stations) to select the closest match of their perceived portion size norms from the provided options for each test food. All participants were blinded to the aim of the study but were reminded of the definition of portion size as ‘the amount of food they eat at one sitting’. A hard copy of the instructions was provided for participants to keep during the session, informed consent was obtained from all participants. Researchers remained in the laboratory room in an unobstructive manner during the study process.

A small token was offered to compensate participants for their time. The study was approved by The University of Sydney Human Research Ethics Committee (ethics approval number 2022/147). Study protocol was registered a priori on the Open Science Framework (OSF registration 10.17605/OSF.IO/X3FM7).

Due to the preliminary nature of this study, a power analysis could not be calculated based on previous literature. Thus, a sample size of 100 participants was used as recommended for preliminary validation in dietary assessment [30].

### Statistical analysis

Data were analysed using IBM SPSS v28 (IBM, Armonk, NY, USA, 2021). Descriptive analyses on participants characteristics were conducted. For each food, data were excluded if participants reported that they did not consume a particular food item. Reported perceived portion size norms from two methods were compared using cross-classification and intra-class correlation coefficients (ICC, two-way mixed model, average measure). Data were classified as a correct match (described as selecting the same image option as the real food), an adjacent match (described as selecting the portion size image one option away from that selected for the real food option), or gross mismatch (described as selecting the portion size image four or more options away from that selected for the real food option) [31]. ICC values < 0.5 were considered poor, 0.5–0.75 moderate, 0.75–0.9 good, and >0.9 excellent [32]. The proportions of over- and underestimation were tested based on real foods being the reference standard. The relationship between cooking confidence and the ability to match images with real foods (that is, the mean proportion of correct match across foods, per participant) was investigated using the Chi-square test.

## Results

### Sample characteristics

A total of 235 subjects passed the screener questionnaire and were invited to the study. A final sample of 114 subjects participated in the validation study. The majority of participants were female (82.5%) with a mean age of 24.8 years and within the normal weight range (77.2%). Details of participant characteristics are presented in Table 2.

**Table 2:** Participants’ characteristics (*n* = 114).

Age, years, mean (SD)	24.8 (8.0)
Gender, females, *n* (%)	94 (82.5)
BMI^a^, kg/m^2^, mean (SD)	21.9 (3.3)
Within normal weight range, *n* (%)	88 (77.2)
Underweight, *n* (%)	13 (11.4)
Overweight, *n* (%)	10 (8.8)
Obese, *n* (%)	3 (2.6)
Physical activity level (PAL)^b^, *n* (%)	
Sedentary	17 (14.9)
Lightly to moderately active	89 (78.1)
Very to extremely active	8 (7.0)
Cooking confidence^c^, *n* (%)	
Low	45 (39.5)
High	69 (60.5)

^a^BMI: body-mass-index, calculated using the formula $\frac{{weight(kg)}}{{height\;2(m)}}$. BMI (kg/m^2^) < 18.5 is underweight, 18.5–24.9 within normal weight range, 25.0–29.9 overweight, >30.0 obese [44].

^b^PAL: estimated using the physical activity factor; classified into three categories as sedentary, lightly to moderately active, and very to extremely active [26, 27].

^c^Cooking confidence: measured using a validated 5-point Likert scale (can cook a nutritious meal; can cook a meal in a short amount of time; can cook spending a lot of money; can follow a recipe); classified as high if participants scored ≥16 out of 20 (very/extremely confident), otherwise as low [29].

Depending on food type, 65 to 111 participants reported consuming this food and selected their perceived portion size norm based on food images and real foods, resulting in a total of 1442 comparisons (Table 3). Cross-classification analysis suggested that overall, 91% of comparisons were classified as a correct or adjacent match, ranging from 86% (crisps) to 97% (nuggets). An average of 53% of all comparisons (ranging from 39% for hot chips to 69% for cola in bottles/cans) achieved an exact match, whilst less than 1% of foods were grossly mismatched (>four categories apart). The ICC of perceived portion size norms between images and corresponding real foods across all food types was good at 0.85 [32]. Good to excellent levels of agreement (ICC above 0.75) were observed in 12 out of 15 test foods, whereas moderate agreement was observed for chocolate bars (ICC 0.71), muffins (ICC 0.69), and banana bread (ICC 0.72).

**Table 3:** Agreement of perceived portion size norms between the food images and corresponding real foods, by food type.

	No. of comparison	ICC (95% confidence interval)^a^	Correct match^b^ %	Correct and adjacent match^b^ %	Gross mismatch^b^ %
Sweet snacks and cakes					
M&Ms	111	0.87 (0.80–0.92)	51	94	0
Chocolate blocks	104	0.92 (0.88–0.94)	60	96	0
Chocolate bar	89	0.71 (0.51–0.82)	58	94	0
Sweet biscuits	98	0.83 (0.67–0.90)	59	92	0
Caramel slices	88	0.76 (0.63–0.84)	50	88	0
Layered cake	104	0.81 (0.72–0.87)	44	87	0
Muffin	95	0.72 (0.46–0.84)	55	92	<1
Banana bread	101	0.69 (0.48–0.80)	51	88	0
Savoury snacks and fast foods					
Savoury biscuits	97	0.83 (0.75–0.89)	48	88	0
Crisps	105	0.80 (0.71–0.86)	44	86	2
Pizza	110	0.93 (0.90–0.96)	57	93	0
Nuggets	97	0.87 (0.77–0.92)	61	97	0
Hot chips	109	0.79 (0.70–0.86)	39	89	0
Sugary carbonated drinks					
Cola cup/glass	69	0.77 (0.53–0.88)	43	93	3
Cola bottle/can	65	0.90 (0.84–0.94)	69	88	0
All	1442	0.85 (0.83–0.87)	53	91	<1

^a^Intra-class correlation coefficient (ICC): ICC estimates and their 95% confidence interval were calculated based on average measures, absolute-agreement, 2-way mixed-effects model.

^b^Correct match described as selecting the same image option as real foods; adjacent match described as selecting the portion size image one option away from that selected for the real food option; gross mismatch described as selecting the portion size image four or more options away that selected for the real food option.

**Fig. 2:**
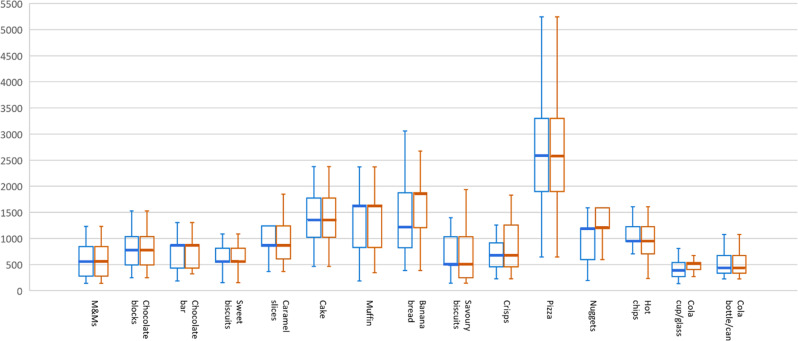
The median energy for perceived portion size norms, images vs real foods, in kJ. Blue boxes indicate reported perceived portion size norms based on real foods; orange boxes indicate reported perceived portion size norms based on images. Thick lines indicate median; upper and lower lines of the box indicate 25th and 75th percentiles, respectively; whiskers above and below the box indicate maximum and minimum values within 1.5 interquartile range above 75th percentile or above 25th percentile; values above 75th percentile or below 25th percentile greater than 1.5 interquartile range were counted as outliers and not shown on this figure.

The median energy for perceived portion size norms varied depending on food type (Fig. 2), ranging from 405 kJ (sugary carbonated drinks in glass/cup) to 2579 kJ (pizza). The median energy reported was similar between food images and real foods for 13 of 15 test foods, but variations in interquartile ranges were observed. For example, portion sizes for banana bread, nuggets and sugary carbonated drinks in glass/cup were overestimated, suggesting the tendency to select larger portion sizes from images than real foods.

The effect of cooking confidence on agreement was analysed using the percentage of correct matches (Table 4). Across all foods, a significant difference between participants with low and high cooking confidence (*p* = 0.04) was observed. Participants with high cooking confidence achieved a significantly higher proportion of correct matches than those with low cooking confidence for four out of 15 foods, including chocolate blocks, chocolate bar, crisps, and hot chips (ps < 0.05). No differences were found for the remaining 11 foods. Regardless of cooking confidence, both under- and overestimations were observed in all test foods. No effect of the presentation order of food images and real foods on the percentage of correct match was observed (*p* > 0.05).

**Table 4:** Frequency of correct match, over-, and underestimation of the reported perceived portion size norms between food images and corresponding real foods, by food type and cooking confidence.

Food type	*P* value^a^	Correct match %		Overestimation %		Underestimation %	
		Low cooking confidence	High cooking confidence	Low cooking confidence	High cooking confidence	Low cooking confidence	High cooking confidence
Overall	0.04*	46	57	36	30	18	14
M&Ms	0.83	49	53	27	32	24	15
Chocolate blocks	0.05*	48	68	29	23	23	9
Chocolate bar	0.03*	43	69	43	28	14	4
Sweet biscuits	0.53	54	62	43	33	3	5
Caramel slices	1.00	49	51	24	25	27	24
Layered cake	0.55	40	47	38	25	22	28
Muffin	0.21	63	49	32	49	8	2
Banana bread	0.84	50	53	40	39	10	8
Savoury biscuits	0.21	40	54	34	22	26	24
Crisps	0.02*	29	55	51	27	20	21
Pizza	0.85	56	58	31	34	13	8
Nugget	0.09	50	68	45	23	5	9
Hot chips	0.02*	25	49	30	23	45	28
SSB cup/glass	0.81	42	46	50	51	8	7
SSB bottle/can	0.41	62	74	23	13	15	13

^a^Differences of % exact match between participants with low and high cooking confidence by Chi-square test; *P < 0.05.

## Discussion

To explore the perceived portion size norm of discretionary foods in Australian adults, an image-based tool for 15 common discretionary foods was developed and validated against corresponding real foods. Agreement between the methods was found to be high based on cross-classification analysis and ICC. Cross classification showed over 90% of selections were matched to the same or adjacent portion size option out of a series of eight options. Similarly, ICC results demonstrated good to excellent agreements for most foods, although three foods had moderate agreement. Cooking confidence was positively associated with level of agreement; participants who reported higher cooking confidence tended to achieve higher percentage of correct matches across foods.

These findings are consistent with other validation studies using a series of food images, with the proportion of correct and adjacent matches varying between 80–98% across studies [31, 33, 34]. Differences of the test foods should be acknowledged as the present study focused on discretionary foods which are usually consumed as between-meal snacks, while previous studies included both recommended food groups and discretionary foods [31, 33, 34]. Variations in the agreement between different methods might be explained by the complexity of the portion size decision process in dietary assessments [35, 36]. Individuals need to make visual perceptions (of volumes) of the displayed amount of food in real-life and relate it to the portion-size tool [34, 35, 37]. Previous research noted that misestimations were common with both over- and underestimations present [31, 34, 38]. It is unclear which factors or which food types lead to over- or underestimation errors due to the high heterogeneity in study designs [37]. In addition, the effect of cooking confidence on the ability to estimate portion size norms has not been well examined, but a study observed that cooking skills contributed to better diet quality and food literacy [28]. Although our findings suggest that participants with higher cooking confidence achieved better validity results, further study is needed to investigate differences in the actual perceived portion size norm between people with different levels of cooking confidence.

The displayed portion size options were carefully considered in the current study. A wide range of options were displayed to minimise potential social desirability bias and these options were pilot tested in the target population to ensure feasibility [19, 25]. Although the validity of methodologies used to measure norms has not been well studied, the self-selected portion size task using a range of real food options appears to be a well-recognised approach to reflect actual food amounts [2, 39]. However, differences in design with other studies should be acknowledged, with many previous studies using matching tasks where food images and corresponding real foods were presented simultaneously [31, 33, 34, 38]. In contrast, participants in the present study were required to conceptualise their portion size norms based on past experience, then select the closest match by using two different methods (real foods versus images) at separate time points within the same session. Higher cognitive ability and memory may be required in the present study to accurately recall portion sizes from day-to-day life [37].

Despite the overall good level of agreement, the images-series for a few foods including banana bread, muffin, and chocolate bar showed only moderate ICCs. For banana bread and muffin, one study noted similar results that the image-series for bread slice performed poorly [31]. One possible explanation could be that serving sizes of cakes vary grossly across settings in Australia (for example, supermarkets and cafés) [40], and consumers have been exposed to a wide range of serving size options. This may add to estimation bias as participants may find generalisation of perceived portion size norm more challenging, especially when using two-dimensional images that are not life-sized [31, 41]. The image series of chocolate bars has not been tested in other validation studies but could potentially be due to small weight increments between the first three options (half fun-size bar 10 g, one fun-size bar 17 g, half standard-size bar 23 g) [31]. Some food characteristics such as the fillings or layers in a chocolate bar may not be easily distinguished from the images [21].

Several strengths of the methodology used to validate this newly developed tool can be noted. This online image-based tool has a low respondent burden as it is easily accessible with any electronic device [21]. The size of increments between portion size options were selected based on package sizes available in Australian supermarkets and food outlets to reflect real food environments. Food images were presented in an ‘animated’ way to mimic a real-world portion size selection process. A reference object and some typical package sizes were included to assist portion size estimation [19]. A sufficiently large sample size recommended for validation studies was recruited [30], and pilot testing was conducted to ensure usability [19]. Despite this, we acknowledge several limitations. The way test foods were presented on white plates may not reflect how consumers eat snacks in real life. As the estimation of perceived portion size norms using real foods and the computer digital images were completed in one session, there is a chance that the first method influenced the second (for example, participants remembered they had selected the smallest size each time). This was minimised by randomising the order of method and foods presented, and no effect of the presentation order on agreement was found. In addition, participants were unaware that the portion size options between the food images and real foods were the same. The convenience sample recruited around university was primarily young, female, and had a high education level, which is not representative of the general population. The proportion of individuals within the normal weight range was higher in this study compared with the population average [42]. However, previous research noted that although females tended to select smaller amount of foods as their ideal portion sizes, gender and BMI are not significantly associated with the ability to quantify food [43]. Future studies could further explore the potential moderating effects of participant characteristics such as age, gender, socioeconomic status, and education level on the perceived portion size norm. There is also potential for expanding the tool to include healthier food types such as vegetables and grains.

## Conclusion

This novel image-based tool developed to estimate perceived portion size norms of discretionary foods showed good agreement with real foods. This tool may be valuable for investigating perceived portion size norms of common discretionary foods in a variety of eating contexts. A better understanding of current portion size norms could be used to inform the development of public health messages and food labelling. It can also help guide the food industry to provide serving sizes more aligned with consumers’ norms and nudge towards smaller sizes over time. This could help create food environments that encourage consumers to select more appropriate discretionary food portion sizes to prevent the overconsumption of these foods.

## Supplementary information

Supplementary material

## Data Availability

All data generated or analysed during this study are included in this article and its supplementary information files.
