# Supplementary material for: Development and validation of an online tool to assess perceived portion size norms of discretionary foods
Source: Eur J Clin Nutr. 2023 May 22;77(8):815–22. doi: 10.1038/s41430-023-01290-y (PMC10393777; doi:10.1038/s41430-023-01290-y)
Supplement: Supplementary file 1 — Supplementary material [file 41430_2023_1290_MOESM1_ESM.docx]

**Supplementary material**

**Appendix 1. Methodology supplementary information**

Criteria for deciding the sizes of each portion size options

The following criteria were used to develop the portion size options for each food. 1) One of the three middle portion sizes (images 3-5) is an estimate of the typical portion sizes based on the latest national nutrition survey (NNPAS 2011-12) (1). 2) For each food, the lower and upper limit of portion size options were guided by searches of available package sizes on chain supermarket and fast-food outlet websites, the interquartile range of typical portion sizes from previous literature (1), as well as a pilot study conducted in March 2022. Considering the food unit bias (that is, people tend to rely on the number of food units rather than the actual serving size when selecting portion sizes) (2), commonly available package and serving sizes were included in portion size options to reflect popular size options in the real-life food environment. When applicable, food packages were presented together with the portion size option (for both images and real foods) as a visual cue for portion size estimation. 3) The increase between portion size options was carefully considered, and the interval between each option varied in weight by at least 15% (20-35% for most options) to ensure the difference between portion sizes was easily distinguished.

Survey design

Eating context was indicated to minimise confusion, the addition of ‘as a side dish’ was provided for nuggets and hot potato chips; the addition of ‘as a main meal was provided for pizza, the addition of ‘between meals’ was provided for drinks, the addition of ‘as a mid-meal snack’ was provided for chocolate cake, banana bread, caramel slice, and muffin; the addition of ‘as a snack’ was provided for the rest of test foods. The marker was centred for all sliding scale questions. Participants were required to click or drag the marker in each question to their corresponding answer before proceeding to the next question.

Preparation for food images and real foods

Thirteen of 15 test foods were photographed on white ceramic plates (25 cm diameter). Two drinks (in glasses/cups and bottles/cans) were photographed in 350 mL glasses. All photographs were taken at an angle of 45 degrees using a smart phone with high-resolution digital camera. A lightbox with neutral colour background was used to eliminate shadows and keep lighting constant. An everyday reference object (local transportation card, credit card size) was displayed next to plate. Foods that required cooking (including pizza, hot chips, and nuggets) were prepared immediately before photographing. All portion sizes were weighed twice on a kitchen scale with 1-gram increments to ensure accuracy.

Real foods were weighed and prepared on the day of the laboratory session using the same method; kitchen scales were calibrated against the scale used to prepare food images to ensure accuracy. All test foods were presented in consecutive order on food stations, labelled with numbers 1-8. Foods were presented on 25-cm diameter white paper plates and drinks were presented in the same 350 mL glasses as per images. One difference in presentation compared with the images was the portion size options for pizza. The food station presented one full pizza evenly cut into eight slices and participants were asked to indicate the number of pizza slices they normally ate using the sliding scale.

Pilot study

A pilot study was conducted in March 2022, with volunteers (n=12) invited to review the image-series and provide feedback regarding the ease of use of sliding scale questions, the portion size options provided, whether they considered people from the same age and gender group could select their normal portion sizes from the images, and how easy/hard to distinguish the amount of food depicted in adjacent images. Portion size options and the sliding scale were then modified according to their feedback. This included changes to portion size options of hot chips, savoury biscuits, M&Ms, and cola in bottles to ensure differences between options were easily distinguished, the addition of eating context in each question, as well as the addition of a demonstration page at the beginning of each section.

Table S1 Portion size weights of food and drink options, in gram

|  | 1 | 2 | 3 | 4 | 5 | 6 | 7 | 8 |
| --- | --- | --- | --- | --- | --- | --- | --- | --- |
| M&Ms | 7 | 14 | 28 | 42 | 61 | 88 | 128 | 180 |
| Chocolate block | 11 | 22 | 34 | 46 | 68 | 90 | 112 | 136 |
| Chocolate bar | 10 | 17 | 23 | 46 | 69 | 92 | 115 | 140 |
| Sweet biscuits | 8 | 14 | 28 | 42 | 56 | 69 | 83 | 98 |
| Caramel slice | 20 | 33 | 46 | 67 | 100 | 142 | 168 | 210 |
| Layered cake | 34 | 65 | 100 | 127 | 170 | 228 | 286 | 374 |
| Muffin | 12 | 22 | 53 | 105 | 152 | 196 | 230 | 305 |
| Banana bread | 31 | 67 | 100 | 155 | 184 | 221 | 252 | 290 |
| Savoury biscuits | 7 | 12 | 25 | 51 | 68 | 94 | 130 | 174 |
| Potato crisps | 10 | 20 | 30 | 40 | 60 | 84 | 122 | 176 |
| Pizza | 62 | 118 | 182 | 247 | 316 | 374 | 442 | 503 |
| Nuggets | 20 | 60 | 120 | 160 | 240 | 320 | 400 | 480 |
| Hot chips | 25 | 50 | 75 | 100 | 130 | 170 | 221 | 287 |
| Cola cup/glass | 75 | 150 | 225 | 300 | 375 | 450 | 525 | 600 |
| Cola bottle/can | 125 | 188 | 250 | 300 | 375 | 600 | - | - |

Table S2 Energy of food and drink options, in kJ^a^

|  | 1 | 2 | 3 | 4 | 5 | 6 | 7 | 8 |
| --- | --- | --- | --- | --- | --- | --- | --- | --- |
| M&Ms | 141 | 283 | 566 | 848 | 1232 | 1778 | 2586 | 3636 |
| Chocolate block | 248 | 495 | 765 | 1035 | 1530 | 2025 | 2520 | 3060 |
| Chocolate bar | 189 | 321 | 435 | 869 | 1304 | 1739 | 2174 | 2646 |
| Sweet biscuits | 155 | 272 | 543 | 815 | 1086 | 1339 | 1610 | 1901 |
| Caramel slice | 370 | 611 | 851 | 1240 | 1850 | 2627 | 3108 | 3885 |
| Layered cake | 463 | 885 | 1361 | 1728 | 2314 | 3103 | 3892 | 5090 |
| Muffin | 186 | 341 | 823 | 1630 | 2359 | 3042 | 3570 | 4734 |
| Banana bread | 376 | 812 | 1212 | 1879 | 2230 | 2679 | 3054 | 3515 |
| Savoury biscuits | 144 | 247 | 515 | 1051 | 1401 | 1936 | 2678 | 3584 |
| Potato crisps | 229 | 458 | 687 | 916 | 1374 | 1924 | 2794 | 4030 |
| Pizza | 647 | 1232 | 1900 | 2579 | 3299 | 3905 | 4614 | 5250 |
| Nuggets | 221 | 662 | 1325 | 1766 | 2650 | 3533 | 4416 | 5300 |
| Hot chips | 236 | 472 | 708 | 944 | 1227 | 1605 | 2086 | 2710 |
| Cola cup/glass | 135 | 270 | 405 | 540 | 675 | 810 | 945 | 1080 |
| Cola bottle/can | 225 | 338 | 450 | 540 | 675 | 1080 | - | - |

^a^Energy calculated based on nutrition information panel on food packages (if available) and the Australian Food Composition Database (3).

References

1. Zheng M, Rangan A, Meertens B, Wu JHY. Changes in Typical Portion Sizes of Commonly Consumed Discretionary Foods among Australian Adults from 1995 to 2011-2012. Nutrients. 2017;9(6).

2. Kerameas K, Vartanian LR, Herman CP, Polivy J. The effect of portion size and unit size on food intake: Unit bias or segmentation effect? Health Psychol. 2015;34(6):670-676.

3. Australian Food composition Database [Internet]. FSANZ. 2022. Available from: https://www.foodstandards.gov.au/science/monitoringnutrients/afcd/pages/default.aspx.
